# Influence of Synthetic Fibers on the Flexural Properties of Concrete: Prediction of Toughness as a Function of Volume, Slenderness Ratio and Elastic Modulus of Fibers

**DOI:** 10.3390/polym15040909

**Published:** 2023-02-11

**Authors:** Alexandre Almeida Del Savio, Darwin La Torre Esquivel, Flávio de Andrade Silva, Joaquín Agreda Pastor

**Affiliations:** 1 Civil Engineering Department, Universidad de Lima, Lima 15023, Peru; 2 Department of Civil and Environmental Engineering, Pontifícia Universidade Católica do Rio de Janeiro, Rio de Janeiro 22451-040, Brazil

**Keywords:** fibers, synthetic macrofibers, polypropylene macrofibers, post-cracking behavior, toughness, residual strength, ASTM C1609

## Abstract

The construction industry requires concrete with adequate post-cracking behavior for applications such as tunnels, bridges, and pavements. For this reason, polypropylene macrofibers are used, which are synthetic fibers that fulfill the function of providing residual strength to concrete. In this study, an experimental plan is carried out to evaluate the bending behavior of concrete reinforced with polypropylene fibers using the four-point bending test according to ASTM C1609. Three fiber dosages (3.6, 7.2 and 10.8 kg/m^3^) and three fiber lengths (40, 50, and 60 mm) were used. The use of macro polypropylene fibers increased the post-cracking behavior of concrete. In addition, based on the experimentally obtained results and available literature data, a multivariable equation was developed to predict the concrete toughness as a function of the volume, slenderness, and modulus of elasticity of the fibers. A Pearson’s correlation coefficient, r of 0.90, showed a strong correlation between the developed equation and the experimental data. From this equation, it was possible to determine the participation of the following parameters in calculating toughness. The participation or weight of the fiber’s modulus of elasticity on the concrete’s tenacity is 26%, the volume of the fiber is 39%, the slenderness is 19%, and the reinforcement index is 16%.

## 1. Introduction

Concrete is the most used construction material in the world [1]. Nowadays, the construction industry requires the development of concrete with improved qualities [2]. Constructing tunnels, bridge decks, and slabs require concrete with high post-cracking strength [3,4,5]. This behavior can be measured through toughness or energy absorption capacity [6]. However, due to its ceramic nature, concrete is a quasi-brittle material and prone to cracking when loads are applied [7]. To improve this property, fibers are incorporated into the mixture to control crack width and propagation [8]. Recently, the Fib Model Code 2010 was developed to design steel fiber-reinforced concrete. However, this code requires beam testing to BS EN 14651 [9]. While this is an adequate design procedure, engineers need a tool that can predict the toughness of steel and synthetic fiber-reinforced concrete without the need for laboratory testing.

Synthetic fibers are artificial fibers that have resulted from research and development in the petrochemical and textile industries [10]. Carbon fibers [11], aramid [5], polyethylene [12], nylon [13], polypropylene [14], polyolefin [15], and polyvinyl alcohol [16] are some kinds of synthetic fibers used as reinforcement in concrete [13]. Due to their nature, synthetic fibers can resist long-term alkaline concrete environments [17,18]. For this reason, these fibers are more resistant to oxidation [19]. Also, according to their geometry, these fibers are small (length 10–75 mm), and their direction is randomly distributed inside concrete [20]. Due to their size, synthetic fibers can be classified into macrofibers and microfibers [11]. Microfibers (less than 0.3 mm diameter) minimize the cracking originating plastic and drying shrinkage but do not provide structural capacity [21]. On the other hand, macrofibers (equal to or greater than 0.3 mm diameter) are used to improve the post-cracking performance of concrete elements subjected to flexural and tensile strength [21]. Polypropylene (PP) macrofibers are the product of an extrusion process where the matrix is rectangular [17]. PP macrofibers have many advantages, such as high tensile strength (250–1000 MPa), chemical stability, and strong adhesion to concrete [12,22]. Furthermore, this type of macrofiber presents a simple manufacturing process and requires less production cost [8,10]. Mechanical strength in concrete increases as long as more PP macrofibers are used [12]. PP macrofibers considerably reduce the cracking due to plastic and drying shrinkage [4,20]. In addition, due to their elastic and inelastic deformation capacity, they are flexible and ductile [10].

PP macrofibers have a low modulus of elasticity, 3–10 GPa, compared to steel, whose modulus of elasticity is between 190–200 Gpa. Bermudez et al. [23] studied the influence of steel fibers and polyvinyl alcohol (PVA)on the shear behavior of ultra-high performance concrete; they found that when steel fibers were used in a volume of 1.5% and PVA fibers at 2.25%, the cut resistance was improved by 3.5 and 2.5 times, respectively. In addition, the steel fibers restricted the appearance of cracks with lower fiber contents concerning the PVA fibers. Chajec and Sadowski [24] found that adding fibers does not significantly affect the unit weight of concrete. In the investigation by Li et al. [25], they found that both steel and polypropylene fibers do not significantly affect the compressive strength of concrete, although steel fibers were more effective. In addition, in the bending tests, the steel fibers were more effective in the pre-peak improvements than in the post-peak ones; while polypropylene fibers had greater effectiveness in the post-peak than in the pre-peak. Rui et al. [26] studied the effect of steel fibers and polyoxymethylene on the characteristics of ultra-high-performance concrete. They found through nanoindentation and X-ray tests that steel fibers improve the modulus of elasticity of the concrete in comparison with synthetic fibers.

Del Savio et al. [27] studied the influence of fiber volume and slenderness on the bending elastic properties and concluded that there is no correlation for low fiber volumes. Abousnina et al. [28] studied the influence of the addition of PP fibers in 40 MPa concrete. They explain that the properties of the concrete matrix dominate the appearance of the initial crack and its propagation to peak load. Dopko et al. considered as variable the fiber’s type, polypropylene (PP), polyvinyl alcohol (PVA), and alkali-resistant glass (ARG) [19] and concluded that greater fiber slenderness (aspect ratio) does not necessarily increase flexural performance in fiber-reinforced concrete since ARG fibers showed a better increase in toughness despite having the lowest slenderness.

Karihaloo and Nallathambi developed an analytical model to determine the toughness of concrete without fibers based on the effective crack model [29,30]. Yap et al. developed an analytical model for the flexural toughness in the cornet’s first crack reinforced with hybrid polypropylene and steel fibers [31]. Wu et al. developed an analytical model to predict the toughness of concrete through the three-point bending test with a groove. This model was based on the model of a fictitious crack [32]. Wang et al. studied the toughness of concrete in the three-point flexure test with the groove by varying the type of groove [33]. Yao et al. developed an analytical solution to calculate initial fracture toughness using the three-point bending test [34,35]. Ramadoss developed a multivariate linear equation for toughness and other properties of steel fiber-reinforced concrete [35].

Although several studies have tested synthetic fibers and their different properties that contribute to concrete, no strong arguments supported by statistical evidence were found about the influence of fibers on the elastic and post-cracking properties of concrete subjected to flexural loads. Mainly because there are more studies about post-cracking properties than the influence of fibers on elastic properties. In addition, it was found that the research that studies post-cracking properties does not develop predictive equations of toughness as a function of the addition of fibers. For this reason, the main contributions of this study are the following: (1) first, a detailed study of the influence of volume and slenderness of PP fibers on the elastic and inelastic properties; and (2) second, a predictive equation of toughness as a function of volume, slenderness, and modulus of elasticity of the fibers is developed, comparing the results of this research with the results of other researchers using synthetic fibers with a different modulus of elasticity and comparing the predictive models using statistical tools. The experimental plan was developed using three fiber dosages (3.6, 7.2 and 10.8 kg/m^3^ and three measurements of fiber slenderness (47, 58, and 70). To have a solid statistical study, 8 beams per dosage were tested. The test performed was the 4-point bending test according to ASTM 1609 standard [6]. 

## 2. Experimental Program

The experimental program was executed at the Structures Laboratory of the University of Lima (Lima, Peru). The description of the materials, mix design, specimens, casting process, tests, and hypothesis tests on the results are presented below.

### 2.1. Materials

The materials used to manufacture the fiber-reinforced concrete were cement, water, coarse aggregate, fine aggregate, superplasticizer, and polypropylene fibers. The cement used was type I Portland cement manufactured according to ASTM C150 [36]. In addition, it has a density of 3120 kg/m^3^ and a specific area of 336 m^2^/kg. The amounts of the chemical components are as follows: tricalcium silicate (C_3_S) at 54.2%, dicalcium silicate (C_2_S) at 11.9%, tricalcium aluminate (C_3_A) at 10.1%, and tetracalcium ferroaluminate (C_4_AF) at 9.7%. 

**Figure 1:**
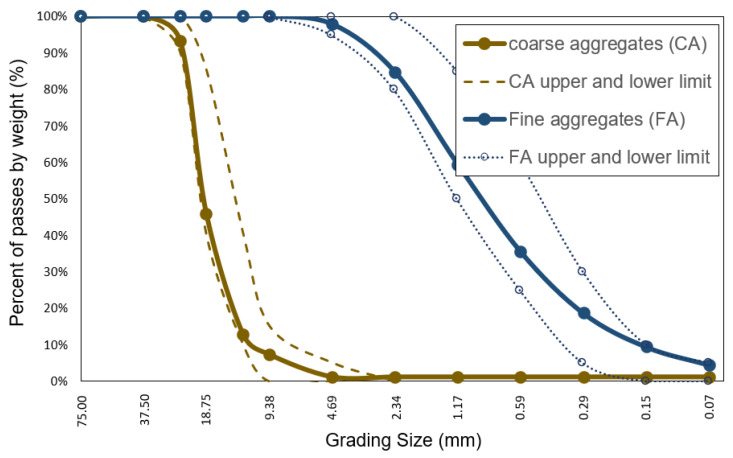
Granulometric curve of aggregates.

Coarse crushed aggregate classified in HUSO N°56 according to ASTM C33 standard was used [37]. This granulometry is characterized by having a maximum nominal size of 25.4 mm with a predominant presence of aggregates with sizes around 18.75 mm and 12.7 mm. The fine aggregate was natural river sand with a fineness modulus of 2.95. The properties of the aggregates can be seen in Table 1, and the granulometric curves in Figure 1.

**Figure 2:**
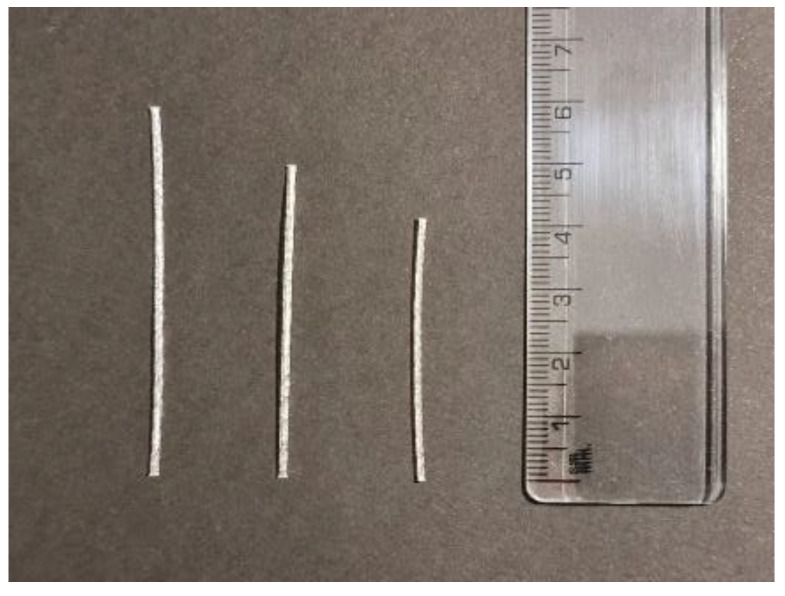
Straight polypropylene fibers with knurled texture.

The fibers used were PP (Fibras Cortadas y Monofilamentos SAC, Lima, Peru) with a tensile strength of 540 MPa and a modulus of elasticity of 4.70 GPa. Fiber density was 920 kg/m^3^. They had a straight shape and a knurled texture. Three types of fibers were used (40, 50, and 60 mm) with a common diameter of 0.86 mm. The resulting slenderness (aspect ratios) were 46, 58, and 70, respectively. The fibers used can be seen in Figure 2 in more detail.

The admixture (Sika, Lima, Peru) used was a high-range water reducer and retarder type G, according to ASTM C494 [38]. The additive has a density of 1200 kg/m^3^, according to the manufacturer’s data. 

### 2.2. Mix Design

The concrete was designed to achieve a compressive strength of 40 MPa at 28 days and a slump of 100 mm. The design method used was the ACI method [39]. The water-cement ratio used was 0.45. The paste-aggregate ratio was 0.45. The percentage of fine aggregate concerning the total aggregates was 45%; the percentage of coarse aggregate was 55%. High-range water-reducing admixture was 1.40% of the weight of cement. Finally, PP fibers (40, 50, and 60 mm in length) were placed in dosages ranging from 0.40%, 0.80%, and 1.20%. A standard mix design was used as a reference for all the designs. Table 2 shows the dosages of the 10 mixtures made. 

### 2.3. Specimens

**Figure 3:**
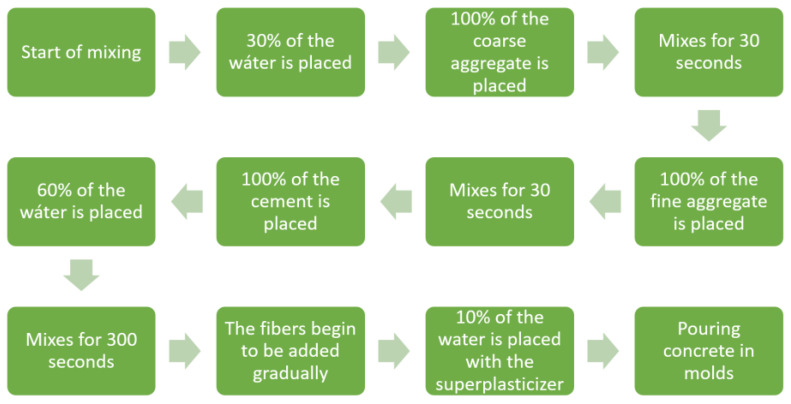
Sequencing of activities for concrete mixing.

The specimens were prepared for the standard test method for the flexural performance of fiber-reinforced concrete (using a beam with four-point loading) according to ASTM C1609 [6]. Parallelepiped concrete beams with dimensions of 150 mm base, 150 mm height, and 550 mm length were prepared. Eight specimens were prepared for each mixture and tested at 28 days. The casting diagram is shown in Figure 3. 

**Figure 4:**
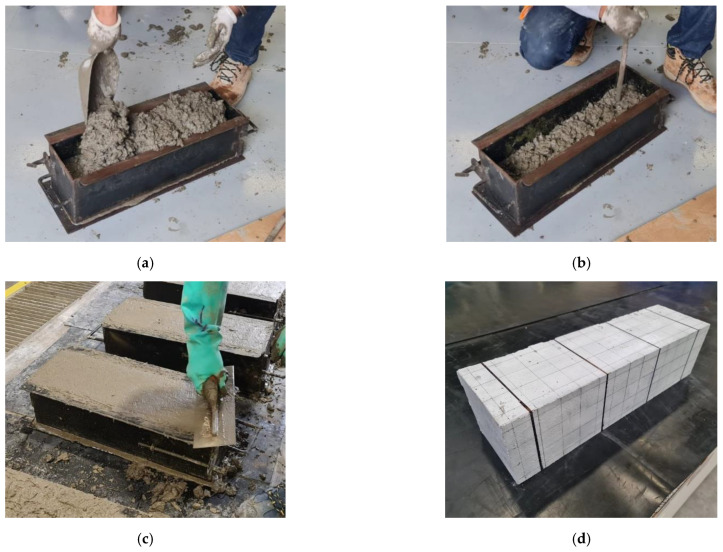
Beam pouring process: (**a**) concrete placement, (**b**) rodding, (**c**) furring and stripping shuttering of the beam, and (**d**) finished specimen.

The freshly mixed concrete was placed in metal molds. The mixture was placed in two layers filling the mold from one direction to the other, Figure 4a, and then compacted with rods, Figure 4b. After repeating this process three times, the concrete was screeded with a trowel, Figure 4c. The dispersion of the fibers is the result of the process executed. Finally, the next day it was demolded and placed in a curing room for 28 days, Figure 4d.

### 2.4. Experimental Method

**Figure 5:**
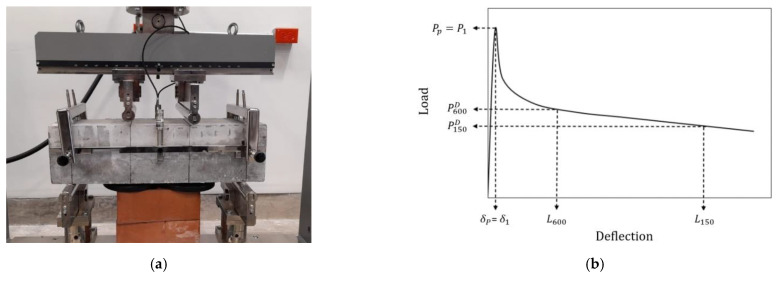
(**a**) Test set-up and (**b**) resulting curve [6].

The test was performed following the ASTM 1609 standard, four-point bending test. This test consisted of supporting a beam at the ends, with a distance between supports of 450 mm, and applying loads with two rollers in the central thirds. The spacing between the rollers applying the load was 150 mm. The load application speed was 0.05 mm/min, controlling the deflection of the beam. A 350 kN capacity compression frame with displacement control was used for the test. The test setup is shown in Figure 5a. The curve resulting from this test is shown in Figure 5b. From this curve, two important post-cracking properties of the concrete can be calculated: toughness and residual strengths. The toughness (*T*) is calculated as the area under the curve with a deflection up to *L*/150, which corresponds to 3 mm, see Figure 5a. The equivalent residual strength $f_{e,150}^{D}$ is the strength the fibers provide in the cracked section through the bridging effect, calculated through Equation (1). An alternative method of calculating the residual strength is calculating the stress that produces the load corresponding to the deflection *L*/150 (*f_res_*), calculated through Equation (2). In this study, the residual strength is determined using the second method. In addition to the flexural tests on concrete, the slump test, according to ASM C143 [40]; the compressive strength test, according to ASTM C39 [41]; and the modulus of elasticity test, according to ASTM C469 [42], were performed.


(1) $$f_{e,150}^{D}=\frac{150\times T}{b\times d^{2}}$$

(2) $$f_{res}=\frac{P\times L}{b\times d^{2}}$$

### 2.5. Hypothesis Testing

Pearson’s hypothesis test was performed to verify the statistical validity of the correlations between variables. The *p*-value, significance level, and the r-value, correlation coefficient, are the results of Pearson’s test. The significance level indicates the probability of being wrong. For concrete material with a high coefficient of variation, it is convenient to set this value as 0.100, as performed in previous studies [27,43].

## 3. Results

Table 3 shows the control properties of concrete in the fresh state, slump and unit weight, compressive strength, and modulus of elasticity in the hardened state. It is observed that the resulting slump shows a similar trend according to previous research [25,44,45,46,47,48,49,50]. The higher the reinforcement index, the lower the slump or slump of the concrete. The average unit weight is 2276.18 kg/m^3^, the average compressive strength is 41.66 MPa, and the average modulus of elasticity is 30.22 GPa.

**Figure 6:**
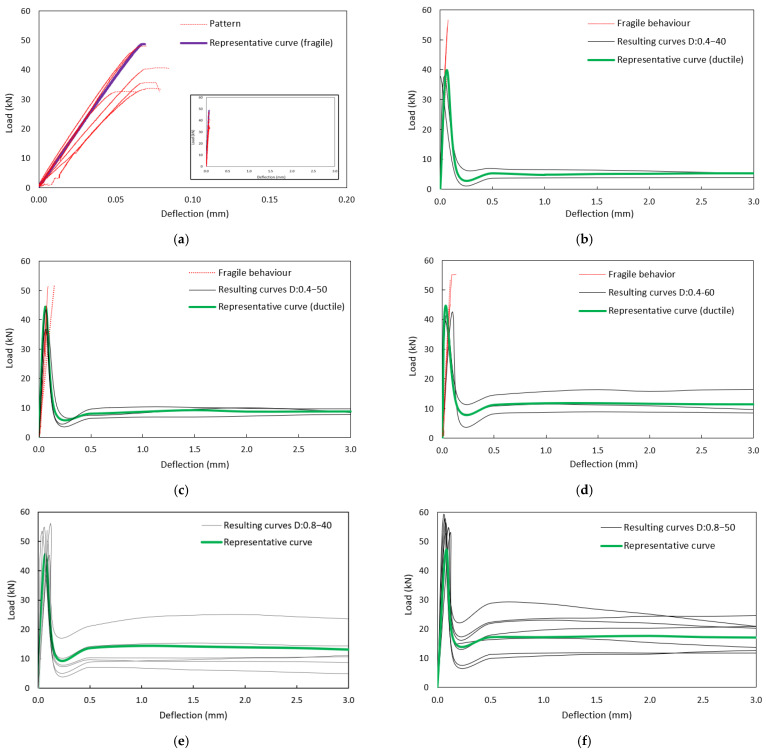

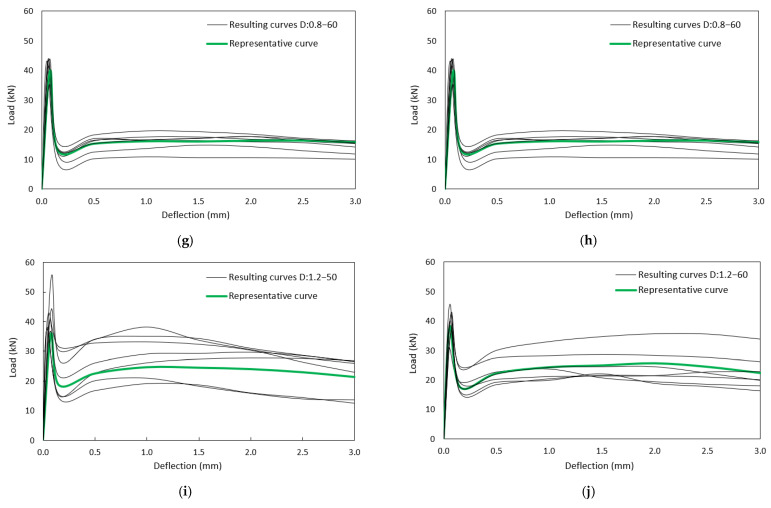
Deflection vs. load curves obtained from bending tests on specimens with different dosages and lengths: (**a**) Pattern, (**b**) D:0.4–40, (**c**) D:0.4–50, (**d**) D:0.4–60, (**e**) D:0.8–40, (**f**) D:0.8–50, (**g**) D:0.8–60, (**h**) D:1.2–40, (**i**) D:1.2–50, (**j**) D:1.2–60.

Figure 6 shows the resulting load vs. deflection curves of all the tests performed for the different mixtures. The curves with red lines represented specimens with brittle failure and did not develop inelastic energy after peak rupture. The curves with black lines represent the specimens with ductile failure, and representative curves for a family of assays are shown in green. These curves were previously corrected because the original curves had instabilities. According to [51], the instabilities are influenced by the type of circuit used in the machine (closed or open), when using a low volume of fibers, and a lack of rigidity of the equipment. 

On the other hand, [52] shows that the rigidity of the machine and the system control methodology influence post-cracking instability. Two actions were taken to correct the curve: (1) the unstable part was eliminated and not considered in the calculation, and (2) because in almost all cases, the post-peak behavior had a constant slope, this line was extrapolated until reaching 3 mm, because by eliminating the unstable part, part of the curve was lost. 

**Figure 7:**
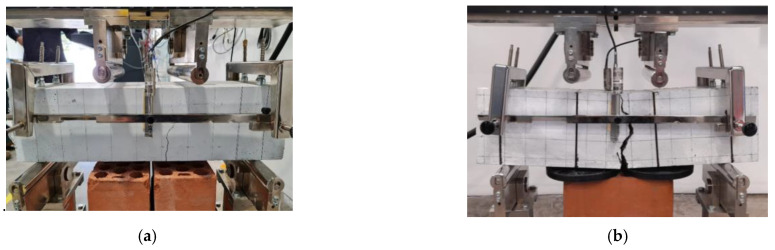
Failure types: (**a**) ductile failure and (**b**) brittle failure.

**Figure 8:**
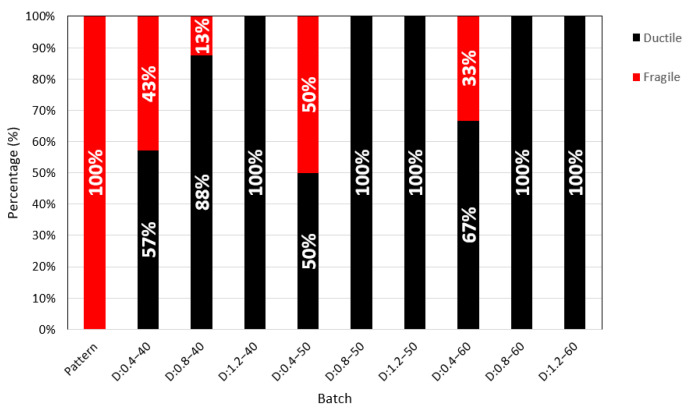
Percent of occurrence of failure modes.

Two failure modes occurred: ductile failures and brittle failures, as shown in Figure 7a,b. Figure 8 shows the percentages of the failure modes that occurred. It is observed that all specimens without fiber had brittle failures. Specimens with a PP fiber dosage of 0.4% exhibited brittle failures in the order of 43, 50, and 33%, with an average of 42%. Specimens with fiber dosages of 0.80% and 1.20% had ductile failures. In general, it is observed that specimens with a reinforcement index greater than 40 present a ductile performance. Manfredi and de Andrade [14] realized a comparative study of toughness test methods and used three dosages of PP fibers (0.33, 0.66, and 1.09%). They concluded that the test based on ASTM 1609 does not properly show the post-cracking mechanical responses of concrete with low fiber volumes because this method’s deflection is less susceptible to measuring crack opening.

Table 4 shows the results of the flexural mechanical properties of reinforced concrete with PP fibers. The flexural tensile strength, the deflection associated with this last strength, and the absorbed energy are shown as flexural elastic properties. As post-cracking properties, the results of toughness and residual strength are shown up to a deflection of 3 mm.

## 4. Discussion

### 4.1. Properties of Elastic Range 

Table 5 shows the hypothesis tests, 21 in total, between the fiber variables (fiber volume, slenderness, and reinforcement ratio) and the elastic properties in bending (bending tensile strength, the strain related to peak strength, and elastic strain energy). The results show that there is not enough evidence to conclude that there is a correlation between the variables because Pearson’s correlation values (*p*-value) in most cases are greater than 0.100. Therefore, the null hypothesis that establishes no correlation between the variables cannot be discarded. Generally, it is observed that the greater the volume of fibers, the lower the significance level; that is, the lower the probability of error. This behavior is consistent with the research by Del Savio et al. [27] since it shows that for volumes greater than 0.8%, the *p* value tends to decrease. Although Del Savio et al. concluded that there was an increase of 0.30 MPa for every 0.40% increase in fibers, this increase was residual and less than 20%. Yin et al. [53] reported that synthetic fibers have no major effects on the elastic properties, which are dominated by the concrete matrix. Several other authors concluded that adding PP macrofibers between 0.3 to 1.2% had no significant effect on the modulus of rupture in concrete beams (increments less than 8%) [9,11,14,19,54,55]. Concerning the influence of the slenderness of the fibers, unlike the volume, the significance level is more erratic since there is no clear trend of the greater the slenderness, the lower the significance level. This is verified by observing low correlation coefficients (r). Noushini et al. [56] reported that for significant increases in flexural tensile strength, the slenderness must be greater than 100. From these results, it can be deduced that the elastic properties will not be affected by the fiber volume, as long as it does not exceed 1.20%, by the fiber slenderness, as long as it does not exceed 70%, and by the reinforcement index, which is the product of the fiber volume by the fiber slenderness. Therefore, the average value of the tensile strength for the mix of this investigation is 5.98 MPa with an average coefficient of variation of 12.07%. This tensile strength value represents 14.34% of the compressive strength. Del Savio et al. shared this relationship in other investigations and found that the flexural shear strength varies from 11.89 to 15.84% [27]. The deflection associated with the average failure load is 0.07 mm, with a coefficient of variation of 27.01%. In other researchers, Mulheron [55] presents his deflection results between 0.06 to 0.09 mm, and Manfredi and de Andrade Silva [14] show as results of their tests deflection values between 0.10 to 0.12 mm. Finally, the average value of the elastic deformation energy, measured as the area under the load-deflection curve, is 1.72 J with a coefficient of variation of 22.95%.

### 4.2. Post-Failures Mechanical Properties

Table 6 shows the results of Pearson’s hypothesis tests (14 in total) between the fiber variables (volume, slenderness, and reinforcement index) and the post-cracking properties of concrete (toughness and residual flexural tensile strength). The results show a significance level (*p*-value) less than 0.050 in most cases and less than 0.100 for all cases. The correlation coefficients (r) also show very high values. It can be deduced that there is a correlation between fiber volume, toughness, and residual strength. Similarly, there is a correlation between fiber slenderness, toughness, and residual strength. Therefore, there is a correlation between the reinforcement index and both post-cracking properties. These results are evidenced when the concrete reinforced with polypropylene fibers (CRFPP) reaches the maximum cracking load of the specimen, and the material develops a residual strength associated with the ductile behavior of the fibers in the concrete matrix. This is because they begin to resist the tensile forces generated and amplified by the crack opening [57,58]. This behavior is known as the bridging effect of the fibers [59,60].

#### 4.2.1. Toughness

**Figure 9:**
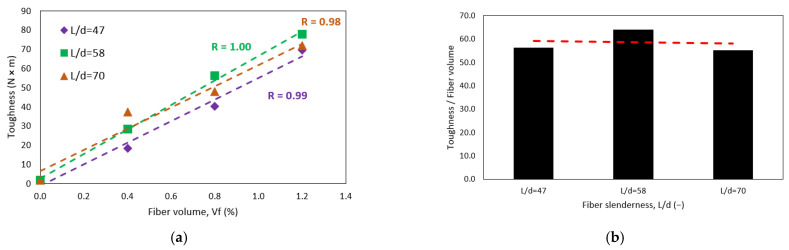
Effect of (**a**) fiber volume fraction on concrete toughness (**b**) the tenacity-fiber volume relationship and slenderness.

Figure 9a shows the relationship between CRFPP toughness and fiber volume dosage. The dashed lines represent the trend of toughness (*T*), obtained from a linear regression for measurements of fiber slenderness of 47, 58, and 70. The correlation coefficients (r) are 0.99, 0.98, and 1.00, respectively. The three trend lines show higher toughness or energy absorption capacity with increasing fiber dosage. Figure 9b compares the slopes of the trend lines. It is observed that the increase in toughness due to an increase in fiber volume is independent of the slenderness of the fibers used. The average rate of increase in toughness is 58.46 J per 1% fiber volume placed. The maximum toughness achieved in this study corresponds to the D:1.2–50 mix with a value of 77.71 J. The average coefficient of variation was 23%. There is no correlation between the coefficient of variation and toughness as observed in the dotted red line.

**Figure 10:**
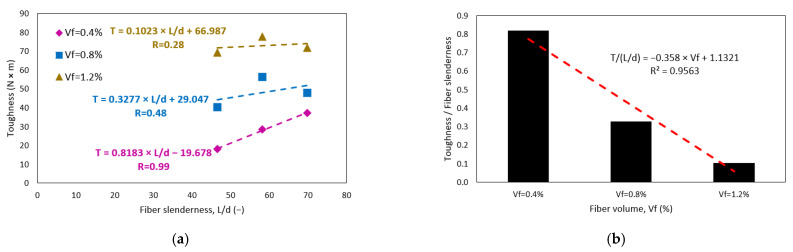
Effect of (**a**) fiber slenderness on concrete toughness (**b**) fiber volume fraction on concrete toughness.

Figure 10a shows the relationship between CRFPP toughness and fiber slenderness. The dotted lines in the figure represent the trend of toughness (*T*), which was obtained from a linear regression for fiber volume of 0.4, 0.8, and 1.2%. In addition, the correlation coefficients (r) are 0.99, 0.48, and 0.28, respectively, when the standard concrete is not considered. These same coefficients are 0.98, 0.96, and 0.95 when the standard concrete is considered in the correlation. This means that there is a marked difference between the toughness of the standard concrete and the concrete with fibers. As the fiber slenderness increases, all three trend lines show higher toughness or energy absorption capacity. Also, when the fiber volume is 1.2%, there is no marked difference whether the fibers are 40, 50, or 60 mm long since the toughness is similar. This can be seen in Figure 10b, which shows the relationship between the slopes of the curves’ tenacity—slenderness concerning the volume of fibers. It can be observed that the slope is higher when the fiber volume is 0.4%, and the slope decreases linearly as the fiber volume increases. The increase in fiber slenderness is more influential when the fiber volume is 0.4% and less influential when the fiber volume is 1.2%. In the ranges studied, the increase in toughness, resulting from the increase in fiber slenderness, per 10 units of slenderness is 8.2, 3.3, and 1.0 J for concretes with fiber volumes of 0.4, 0.8, and 1.2 %, respectively.

**Figure 11:**
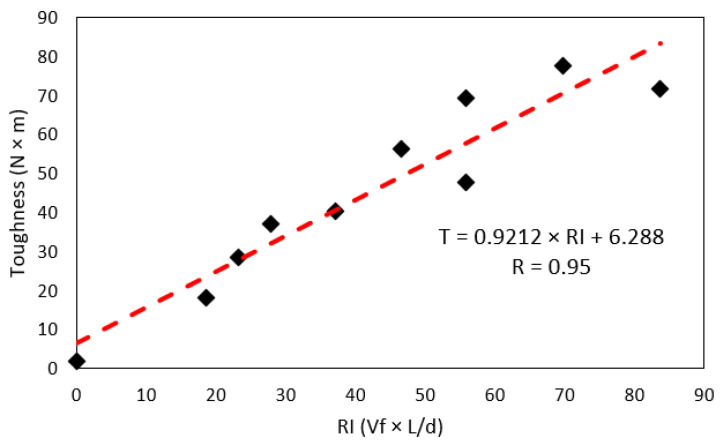
Effect of fiber volume fraction on linear regression of concrete toughness.

An important parameter that includes slenderness and fiber volume is the reinforcing index (*RI*), which represents the product of both variables. Figure 11 shows the relationship between CRFPP toughness and fiber reinforcement index. The dashed lines in the figure represent the trend of toughness (*T*). The *p*-value of this correlation is very close to zero (0.000), so the probability of error is zero. The correlation coefficient (R) was 0.95, classified as strong. The trend line shows that the higher the rate of PP fiber reinforcement, the higher the toughness of the concrete. These results are in accordance with previous research [5,11,14,55]. The results of Conforti [11], Manfredi and de Andrade Silva [14], and Gao [61] are in the range of 24 to 74 J. These results are in accordance with the present investigation since they show toughness ranges between 18 to 78 J. On the other hand, Dopko [19] and LaHucik [5] show relatively low values, between 10 to 30 J. In contrast, tests performed with steel fibers show much higher values than tests performed with PP microfibers [58,62]. Carrillo [58] presents toughness results between 40 to 120 J. Finally, the trend line shows that an increase of 10 *RI* units will produce an increase of 9.2 J in toughness.

#### 4.2.2. Residual Resistance 

**Figure 12:**
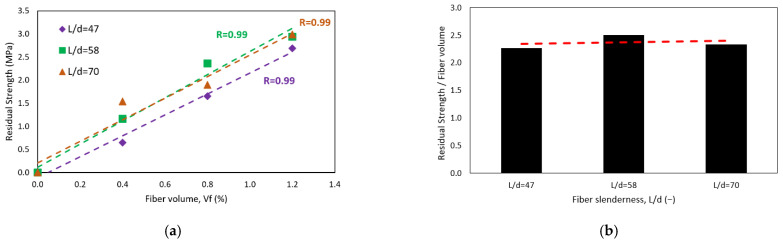
Effect of (**a**) fiber volume fraction on residual resistance of concrete (**b**) the residual strength-fiber volume relationship and slenderness.

Figure 12a shows the relationship between residual strength, measured at 3 mm deflection of CRFPP, and fiber volume dosage. The dashed lines in the figure represent the trend of the residual strength *L*/150 (*f_res_*), which was obtained from a linear regression for measurements of fiber slenderness of 47, 58, and 70. These correlations are statistically significant as their *p*-values are 0.008, 0.009, and 0.024, respectively, all values being less than 0.010. Therefore, the null hypothesis is rejected, and the hypothesis where there is a correlation between the variables is accepted. The correlation coefficient R is 0.99 for all cases. The trend lines show an increase in residual strength. This resistance increases when the volume of the fibers also increases. As with toughness, the increase in residual strength per volume fraction is similar regardless of the fibers’ slenderness. This can be seen in Figure 12b. The average increase in toughness per 1% increase in fiber volume is 2.37 J. Other authors conclude that using PP macro fibers between 0.3 to 1.5 % increases the residual strength of concrete beams [11,19,55]. On the other hand, this research presents results like those of Conforti [11] and Mulheron [54] since the range of residual strength is between 1.15 to 4.29 MPa. In contrast to these tests, Carrillo [57] shows higher results concerning residual strength, results between 1.9 to 6.4 MPa based on the application of steel fibers.

**Figure 13:**
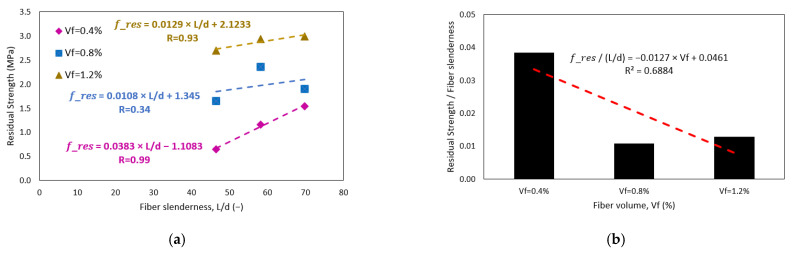
Effect of (**a**) fiber slenderness on residual resistance of concrete (**b**) the residual strength-fiber slenderness relationship and fiber volume.

Figure 13a shows the relationship between CRFPP residual strength and fiber slenderness. The dashed lines in the figure represent the residual strength trend obtained from a linear regression for fiber volume of 0.4, 0.8, and 1.2%. These relationships are statistically significant since the resulting *p*-value is 0.043, 0.060, and 0.027, respectively, considering the standard mixture. The coefficients of determination (R) are 0.99, 0.34, and 0.93, respectively. The trend line with the greatest slope is presented when a fiber volume dosage of 0.4% decreases as the fiber volume increases. This means that the influence of increasing the slenderness of the fibers is greater when the fiber volume is lower, as shown in Figure 13b. The increase in toughness per 10 units of slenderness is 0.38, 0.10, and 0.12 J for volumes of 0.4, 0.8, and 1.2%, respectively.

**Figure 14:**
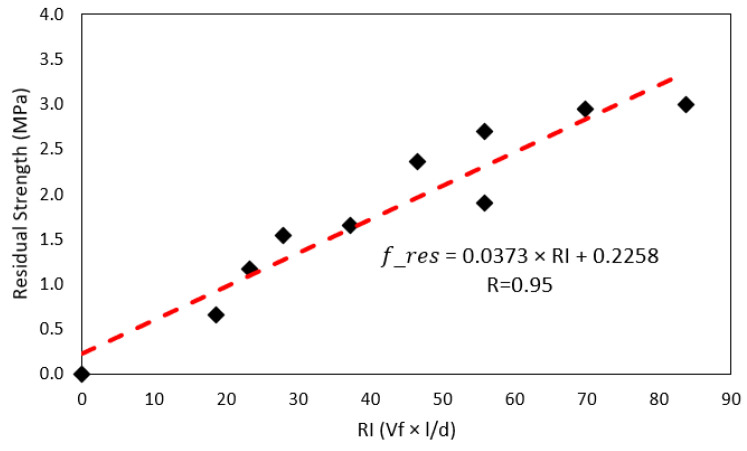
Linear regression of residual strength *L*/150 of concrete according to fiber reinforcement index.

Figure 14 shows the relationship between residual strength and fiber reinforcement index. The dashed lines in the figure represent the trend of the residual strength. This correlation is statistically significant as it has a *p*-value of 0.000, less than 0.100. The correlation coefficient (r) was 0.95. The trend line shows that the higher the reinforcement index of the PP fibers, the higher the residual strength of the concrete. The highest experimental residual strength obtained is 2.99 MPa when a reinforcement index of 83.7 is used, and the lowest residual strength obtained is 0.65 MPa for a reinforcement index of 18.6. As a reference, Dopko [19] presents his residual strength results in the range of 1.48 to 4.72 MPa for pp fibers. Carrillo [58] presents his results in the range of 1.3 to 4.0 MPa for steel fibers. Finally, the trend line shows that an increase of 10 *RI* units will increase 0.4 J in toughness.

**Figure 15:**
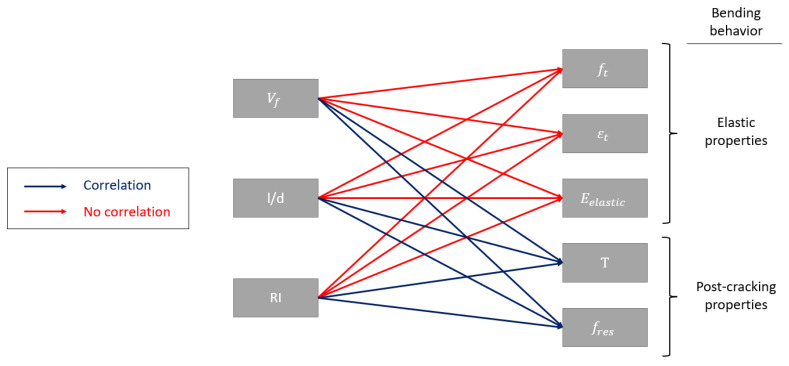
Correlation between synthetic fiber variables and flexural properties.

Figure 15 shows a summary correlation diagram between variables to conclude the correlation study. As shown, volume, slenderness, and fiber reinforcement index do not influence the flexural elastic properties of concrete with PP fibers (lines marked in red). However, they have a marked influence on the post-cracking flexural properties and lines (marked in blue).

### 4.3. Statistical Analysis

#### 4.3.1. Influence of Fiber Stiffness on Concrete Toughness

Information on toughness results with synthetic fibers was collected from other authors. This information is shown in Table 7. The objectives of this research were three: (1) to evaluate the influence of *RI* on the toughness of concrete with synthetic fibers and to compare with the results of the present investigation, (2) to evaluate the influence of fiber stiffness on concrete toughness, and (3) to evaluate the coefficient of variation of toughness tests of other authors.

**Figure 16:**
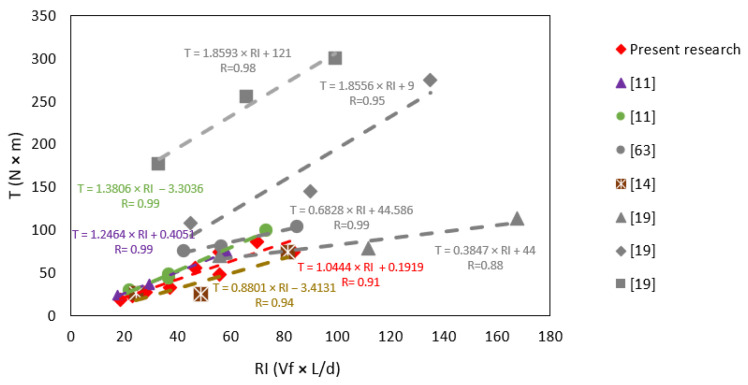
Comparison of toughness results according to *RI*.

**Figure 17:**
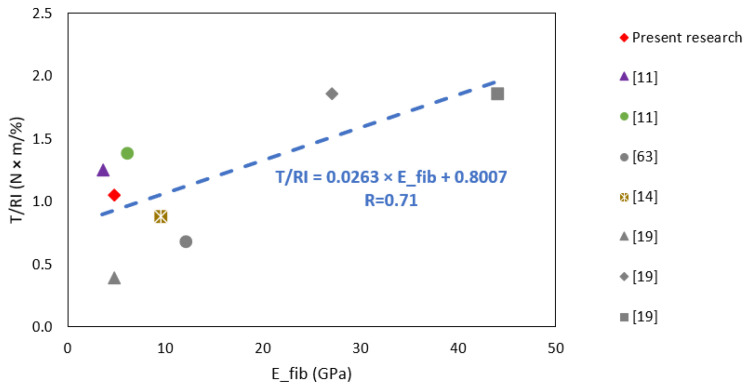
Influence of fibers modulus of elasticity on the slope of the toughness-*RI* curve.

Figure 16 shows the influence of the reinforcement index on toughness for this and other studies. As can be seen, there are high correlation coefficients in all cases, indicating a strong to very strong correlation. On the other hand, some trend lines are steeper than others. The modulus of elasticity of the fibers used explains this. The modulus of elasticity of the present investigation is 4.7 GPa. Conforti et al. [11] work with two types of fiber having a modulus of elasticity of 3.5 and 6.0 GPa, Carmona and Molins [63] work with fibers having a modulus of elasticity of 12 GPa, Dopko et al. [19] work with three types of synthetic fibers having a modulus of elasticity of 4.7, 27 and 44 GPa and, lastly, Manfredi and de Andrade Silva [14] work with fibers having a modulus of elasticity of 9.5 GPa. Figure 17 shows the correlation between the toughness-*RI* curve’s slope and the fibers’ modulus of elasticity. The result shows that there is a correlation. In other words, the higher the modulus of elasticity, the higher the increase in toughness per unit RI. Equation (3) shows the result of the linear regression between these variables.

(3) $$T=RI\times \left(0.026\times E_{fib}+0.800\right)$$

Concerning the coefficient of variation, CV, not many authors present this parameter. From the review made, it was found that all the concretes used were made in the laboratory. The coefficient of variation of this study is 21%. For reference, the coefficient of variation published by Conforti et al. [11] and Manfredi and Silva [14] are 14.4 and 17.3%, respectively. The average coefficient of variation is 17.81%. This result is similar to the one reported for concretes with steel fibers. For example, LaHucik et al. [5] reported a coefficient of variation of 18.36% for steel fiber-reinforced concrete.

#### 4.3.2. Development of Empirical Predictive Equations

**Figure 18:**
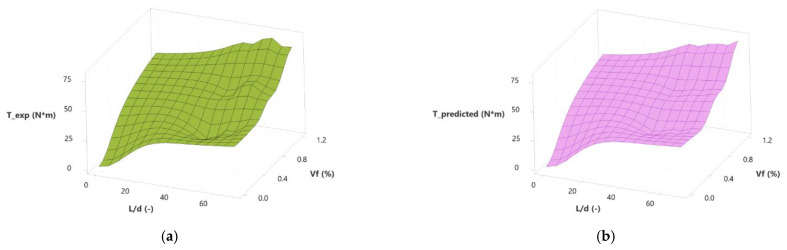
Surface plots of toughness according to slenderness and fiber volume for this investigation (**a**) experimental and (**b**) calculated.

**Figure 19:**
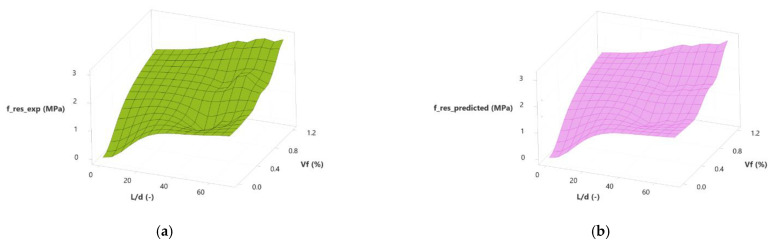
Surface plots of residual tensile strength according to slenderness and fiber volume (**a**) experimental and (**b**) calculated.

To process the data from this investigation, multiple nonlinear regression was used to obtain relationships between fiber-related variables (*Vf* and *L*/*d*;) and mechanical strengths (*T*, *f_res_*). Equations (4) and (5) show the calculation results of (1) toughness as a function of fiber volume and slenderness and (2) residual strength as a function of fiber volume and slenderness. Figure 18 and Figure 19 show the contour surface of these equations. Figure 18a shows the contour surface of the experimental results of the toughness. Figure 18b shows the contour surface calculated with Equation (4). Figure 19a shows the contour surface for the residual strength’s experimental results, and Figure 19b shows the contour surface calculated with Equation (4).

(4) $$T=1.315+34.645\times Vf^{2}+0.387\times \left(L/d\right)$$

(5) $$f_{res}=1.336\times Vf^{2}+0.018\times \left(L/d\right)$$

**Figure 20:**
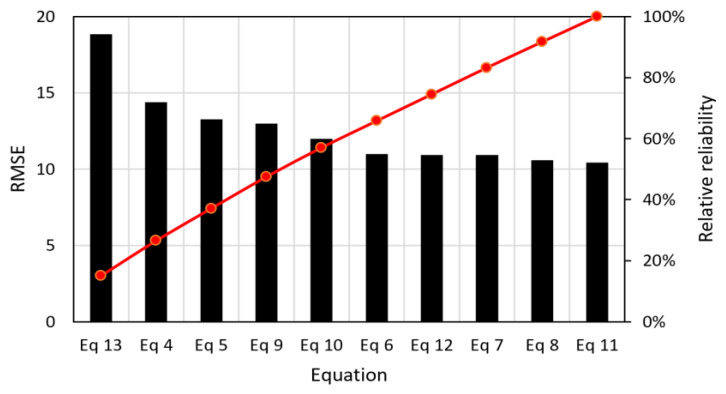
Histogram of RMSE values with the Pareto chart for the different predictive models.

To have a more representative predictive equation, the results of Table 7 were used. A multivariate nonlinear regression was performed, including, this time, the modulus of elasticity of the fibers. Several models were tested using Minitab software (Minitab, version 19.2020.1, 64bit, United States). The results of the analysis can be seen, from Equation (6) to (13). To compare these equations and determine which one is the most convenient, a root-mean-square deviation (RMSD) analysis was performed between the results of the equations and the experimental results. This is shown in more detail in equations. Figure 20 shows the histogram of RMSD values for each equation, including Equations (4) and (5), and the Pareto chart showing the relative reliability between equations is also shown. 

**Figure 21:**
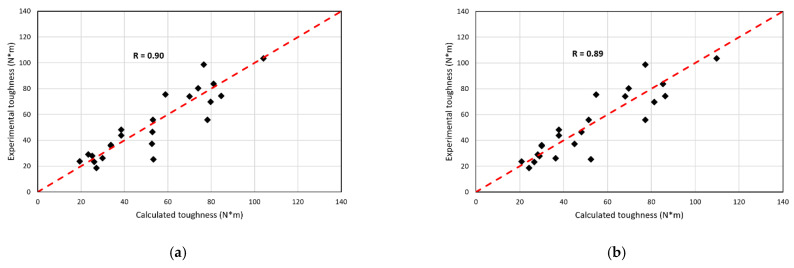
Correlation of calculated and experimental toughness values: (**a**) Equation (11) and (**b**) Equation (12).

The result shows that Equation (11) is the one with the lowest RMSE. Therefore, it has the best fit. However, the equation that also presents a low RMSE value is Equation (12). The authors believe that this equation is the most convenient because it shows the contribution of each variable separately so that the total result is the sum of all the monomials. The equation has no negative monomials, unlike Equations (7), (8) and (11). In addition, the correlation coefficient of Equation (11) is greater than that of 12. The values are very close, 0.90 for Equation (11) and 0.89 for Equation (12). Figure 21a,b show the relationship between calculated and experimental toughness with Equations (11) and (12). In Equation (12), the influence of the modulus of elasticity on toughness is 26%, fiber volume squared is 39%, slenderness is 19%, and the influence of reinforcement index is 16%.

(6) $$T=2.00\times E_{fib}+42.688\times Vf^{2}+0.189\times \left(\frac{L}{d}\right)$$

(7) $$T=10.296+2.15\times E_{fib}+42.065\times Vf^{2}+0.016*Vf\times \left(\frac{L}{d}\right)$$

(8) $$T=23.047+2.126\times E_{fib}+22.203\times Vf^{2}-0.367\times \left(\frac{lL}{d}\right)+0.525\times Vf\times \left(\frac{L}{d}\right)$$

(9) $$T=2.089\times E_{fib}+61.340\times Vf-0.054\times \left(\frac{L}{d}\right)+0.111\times Vf\times \left(\frac{L}{d}\right)$$

(10) $$T=42.422+\left(-19.103+27.349*Vf+0.208\times \left(\frac{lL}{d}\right)-0.278\times Vf\times \left(\frac{L}{d}\right)\right)\times E_{fib}$$

(11) $$T=2.117\times E_{fib}+52.301\times Vf-0.184\times \left(\frac{L}{d}\right)+0.254\times Vf\times \left(\frac{L}{d}\right)$$

(12) $$T=1.916\times E_{fib}+36.128\times Vf^{2}+0.132\times \left(\frac{L}{d}\right)+0.180\times Vf\times \left(\frac{L}{d}\right)$$

(13) $$T=\left(18.315+1.868\times Vf^{2}-0.232\times \left(\frac{L}{d}\right)+0.058\times Vf\times \left(\frac{L}{d}\right)\right)\times E_{fib}$$

## 5. Conclusions

An experimental plan was executed to investigate the influence of volume and slenderness of PP fibers on the elastic and inelastic properties of fiber-reinforced concrete and to predict the value of toughness as a function of volume, slenderness and modulus of elasticity of synthetic fibers. The experimental plan was developed using three fiber dosages (0.4, 0.8, and 1.2%) and three measurements of fiber slenderness (47, 58, and 70).

- The results showed a strong correlation between fiber parameters and post-cracking properties. The higher the volume, slenderness, and reinforcement index of PP fibers, the higher the toughness and residual strength of the concrete. Pearson’s hypothesis supports this since the *p*-values in all correlations between fiber parameters and elastic properties were less than the established significance level of 10%, rejecting the null hypothesis.
- The modulus of elasticity of the fibers conditions the toughness increase due to the reinforcement index. Investigations using fibers with high values of fiber modulus of elasticity reported greater increases in toughness concerning fibers with low values of modulus of elasticity. The rate of increase in toughness concerning *RI* is 0.263 units per 10 GPa increase in fiber modulus of elasticity.
- From the data of the present investigation and other investigations, a multivariable nonlinear equation (Equation (12)) has been developed to determine the toughness of concrete according to volume, slenderness, and fiber modulus of elasticity. This equation presents a correlation coefficient of 0.89, which is considered strong. From this equation, it was possible to determine that the participation of the modulus of elasticity on the toughness is 26%, on the fiber volume squared is 39%, on the slenderness is 19% and on the reinforcement index is 16%.

## Figures and Tables

**Table 1:** Physical properties of aggregates.

Property	Unit	Fine Aggregate	Coarse Aggregate
Maximum diameter	mm	9.52	38.1
Nominal maximum diameter	mm	4.76	25.4
Fineness modulus	–	2.95	7.41
Specific weight of dry mass	g/cm^3^	2.66	2.69
Specific weight of mass SSD	g/cm^3^	2.69	2.72
Specific weight of bulk mass	g/cm^3^	2.73	2.78
Absorption	Percent	1.06	1.15
Loose unit weight	kg/m^3^	1716.46	1465.82
Compacted unit weight	kg/m^3^	1969.75	1610.22

**Table 2:** Design of concrete mixtures using the ACI method (kg/m^3^).

Type	Water	Cement	Fine Aggregate	Coarse Aggregate	Water Reducer	PP Fibres 40 mm	PP Fibres 50 mm	PP Fibres 60 mm
Pattern	226.6	502.8	721.1	891.2	7.1	–	–	–
D:0.4–40	226.6	502.8	721.1	891.2	7.1	3.6	–	–
D:0.8–40	226.6	502.8	721.1	891.2	7.1	7.2	–	–
D:1.2–40	226.6	502.8	721.1	891.2	7.1	10.8	–	–
D:0.4–50	226.6	502.8	721.1	891.2	7.1	–	3.6	–
D:0.8–50	226.6	502.8	721.1	891.2	7.1	–	7.2	–
D:1.2–50	226.6	502.8	721.1	891.2	7.1	–	10.8	–
D:0.4–60	226.6	502.8	721.1	891.2	7.1	–	–	3.6
D:0.8–60	226.6	502.8	721.1	891.2	7.1	–	–	7.2
D:1.2–60	226.6	502.8	721.1	891.2	7.1	–	–	10.8

**Table 3:** Control properties in the fresh and hardened state of concrete.

Batch	*RI* (Vf×L/d)	Slump (mm)	Density (kg/m^3^)	$f_{cr}(\mathrm{MPa})$	*E* (GPa)
Reference	0.0	240	2254.7	42.16	29.94
D:0.4–40	18.4	210	2229.2	41.17	30.66
D:0.8–40	36.8	175	2268.9	42.89	29.95
D:1.2–40	55.2	95	2268.9	39.56	33.20
D:0.4–50	23.2	210	2322.8	39.64	29.03
D:0.8–50	46.4	70	2331.3	41.20	31.14
D:1.2–50	69.6	46	2243.4	42.90	29.09
D:0.4–60	28.0	140	2283.1	42.56	30.28
D:0.8–60	56.0	125	2296.3	40.98	29.16
D:1.2–60	84.0	111	2263.2	43.60	29.75

**Table 4:** Flexural mechanical properties of reinforced concrete with PP fibers.

Batch	Vf (%)	*l/d* (−)	*RI* (Vf×L/d)	*f’c* (MPa)	COV (%)	$f_{t}$ (Mpa)	COV (%)	$\delta_{e}$ (mm)	COV (%)	$E_{e}$ (N×m)	COV (%)	T (N×m)	COV (%)	$f_{res}$ (Mpa)	COV (%)
Reference	0.0	0.0	0.0	42.16	4.71	5.63	16.43	0.07	10.53	1.43	19.35	1.72	13.98%	0	0.83%
D:0.4–40	0.4	46.5	18.6	41.17	3.03	5.89	18.68	0.06	19.80	1.69	26.86	18.130	21.13%	0.65	16.12%
D:0.8–40	0.8	46.5	37.2	39.64	4.11	6.71	9.63	0.08	32.45	2.20	12.61	40.220	40.39%	1.65	47.16%
D:1.2–40	1.2	46.5	55.8	42.56	1.19	6.45	9.23	0.08	29.04	2.18	14.11	69.360	18.15%	2.69	27.99%
D:0.4–50	0.4	58.1	23.3	42.89	4.29	5.80	13.77	0.08	37.61	1.73	20.54	28.400	12.64%	1.16	8.95%
D:0.8–50	0.8	58.1	46.5	41.20	7.43	7.24	7.42	0.08	25.57	2.16	18.28	56.230	27.55%	2.36	26.27%
D:1.2–50	1.2	58.1	69.8	40.98	8.55	5.50	15.52	0.07	31.41	1.54	36.37	77.710	18.19%	2.94	26.59%
D:0.4–60	0.4	69.8	27.9	39.56	2.56	5.89	10.95	0.06	49.29	1.43	44.30	37.160	23.96%	1.54	30.50%
D:0.8–60	0.8	69.8	55.8	42.90	3.60	5.47	7.37	0.06	18.33	1.41	13.76	47.840	14.13%	1.90	15.42%
D:1.2–60	1.2	69.8	83.7	43.60	18.75	5.20	11.74	0.06	16.09	1.46	23.32	71.740	19.91%	2.99	24.78%

**Table 5:** Application of Pearson’s correlation coefficient for elastic properties and fiber variables.

Item	MV	IV	DV	R2	R	*p*	Null Hypothesis
1	*L/d* = 47	Vf	*ft*	73.07%	85.48%	0.145	no rejection
2	*L/d* = 58	Vf	*ft*	2.74%	16.55%	0.834	no rejection
3	*L/d* = 70	Vf	*ft*	49.80%	70.57%	0.294	no rejection
4	Vf = 0.4%	*L/d*	*ft*	0.00%	0.00%	0.998	no rejection
5	Vf = 0.8%	*L/d*	*ft*	46.71%	68.34%	0.521	no rejection
6	Vf = 1.2%	*L/d*	*ft*	91.75%	95.79%	0.188	no rejection
7	–	*RI*	*ft*	2.55%	15.97%	0.660	no rejection
8	*L/d* = 47	Vf	$\delta_{e}$	51.16%	71.53%	0.285	no rejection
9	*L/d* = 58	Vf	$\delta_{e}$	0.03%	1.73%	0.983	no rejection
10	*L/d* = 70	Vf	$\delta_{e}$	59.96%	74.34%	0.226	no rejection
11	Vf = 0.4%	*L/d*	$\delta_{e}$	1.53%	12.37%	0.921	no rejection
12	Vf = 0.8%	*L/d*	$\delta_{e}$	75.00%	86.60%	0.333	no rejection
13	Vf = 1.2%	*L/d*	$\delta_{e}$	98.38%	99.19%	0.081	rejected
14	–	*RI*	$\delta_{e}$	0.77%	8.77%	0.810	no rejection
15	*L/d* = 47	Vf	$E_{e}$	88.39%	94.02%	0.060	rejected
16	*L/d* = 58	Vf	$E_{e}$	9.67%	31.10%	0.689	no rejection
17	*L/d* = 70	Vf	$E_{e}$	61.89%	78.67%	0.213	no rejection
18	Vf = 0.4%	*L/d*	$E_{e}$	63.97%	79.98%	0.410	no rejection
19	Vf = 0.8%	*L/d*	$E_{e}$	78.42%	88.56%	0.308	no rejection
20	Vf = 1.2%	*L/d*	$E_{e}$	83.35%	91.30%	0.268	no rejection
21	–	*RI*	$E_{e}$	0.16%	4.00%	0.914	no rejection

**Table 6:** Application of Pearson’s correlation coefficient for post-failure properties and fiber variables.

Item	MV	IV	DV	R2	R	*p*	Null Hypothesis
1	*L/d =* 47	Vf	*T*	98.42%	99.21%	0.008	rejected
2	*L/d* = 58	Vf	*T*	99.71%	99.85%	0.001	rejected
3	*L/d* = 70	Vf	*T*	95.85%	97.90%	0.021	rejected
4	Vf = 0.4%	*L/d*	*T*	95.19%	97.57%	0.024	rejected
5	Vf = 0.8%	*L/d*	*T*	91.20%	95.50%	0.045	rejected
6	Vf = 1.2%	*L/d*	*T*	91.09%	95.44%	0.046	rejected
7	*–*	*RI*	*T*	89.99%	94.86%	0.000	rejected
8	*L/d* = 47	Vf	$f_{res}$	98.49%	99.24%	0.008	rejected
9	*L/d* = 58	Vf	$f_{res}$	98.13%	99.06%	0.009	rejected
10	*L/d* = 70	Vf	$f_{res}$	95.29%	97.62%	0.024	rejected
11	Vf = 0.4%	*L/d*	$f_{res}$	91.55%	95.68%	0.043	rejected
12	Vf = 0.8%	*L/d*	$f_{res}$	88.35%	93.99%	0.060	rejected
13	Vf = 1.2%	*L/d*	$f_{res}$	94.76%	97.34%	0.027	rejected
14	*–*	*RI*	$f_{res}$	90.88%	95.33%	0.000	rejected

**Table 7:** Toughness results from other research works.

Item	Author	*f’c* (MPa)	$E_{fib}\;(\mathrm{GPa})$	Vf (%)	*l* (mm)	*d* (mm)	*l/d* (−)	RI (Vf×L/d)	T (N×m)	COV (%)
1	[11]	50.00	3.5	0.33	40	0.75	53.3	17.6	23.65	12.0
		50.00	3.5	0.55	40	0.75	53.3	29.3	35.75	18.0
		50.00	6.0	0.33	54	0.81	66.7	22.0	29.00	14.0
		50.00	6.0	0.55	54	0.81	66.7	36.7	43.80	18.0
		80.00	3.5	0.55	40	0.75	53.3	29.3	36.35	17.0
		80.00	3.5	1.10	40	0.75	53.3	58.7	74.05	10.0
		80.00	6.0	0.55	54	0.81	66.7	36.7	48.13	13.0
		80.00	6.0	1.10	54	0.81	66.7	73.3	98.71	13.0
2	[63]	38.60	12.0	0.66	54	0.84	64.3	42.4	75.50	−
		40.90	12.0	0.88	54	0.84	64.3	56.6	80.30	−
		42.30	12.0	1.32	54	0.84	64.3	84.9	103.50	−
3	[19]	−	4.7	0.50	38	0.34	111.8	55.9	70.00	−
		−	4.7	1.00	38	0.34	111.8	111.8	78.00	−
		−	4.7	1.50	38	0.34	111.8	167.6	113.00	−
		−	27.0	0.50	18	0.20	90.0	45.0	108.00	−
		−	27.0	1.00	18	0.20	90.0	90.0	145.00	−
		−	27.0	1.50	18	0.20	90.0	135.0	275.00	−
		−	44.0	0.50	43	0.65	66.2	33.1	177.00	−
		−	44.0	1.00	43	0.65	66.2	66.2	255.00	−
		−	44.0	1.50	43	0.65	66.2	99.2	300.00	−
4	[14]	73.00	9.5	0.33	40	0.54	74.1	24.4	26.22	35.00
		73.00	9.5	0.66	40	0.54	74.1	48.8	25.32	9.00
		73.00	9.5	1.10	40	0.54	74.1	81.4	74.33	8.00

## Data Availability

The data supporting the findings of this study are available within the article.

## Abbreviations

*L*	fiber length (mm)
*d*	fiber diameter (mm)
*L/d*	slenderness (−)
$V_{f}$	fiber volume (%)
*RI*	reinforcing index ($V_{f}*l/d$)
*T*	toughness calculated at 3 mm deflection (N * m)
$f_{e,150}^{D}\;$	residual resistance equivalent (MPa)
$f_{res}$	residual resistance measured at 3 mm deflection (N * m)
$f_{t}$	flexural tensile strength (MPa)
$\delta_{e}$	deflection related to cracking load (mm)
$E_{e}$	elastic strain energy (N * m)
*b*	beam type specimen base (m)
*d*	beam type specimen height (m)
$f_{cr}$	average compressive strength (MPa)
*E*	modulus of elasticity of concrete (GPa)
$E_{fib}$	modulus of elasticity of fibers (GPa)
COV	coefficient of variation (%)
MV	moderator variable
IV	independent variable
DV	dependent variable
R2	coefficient of determination
R	correlation coefficient
*p*	*p*-value
